# Supplementary material for: Iterative improvement in the automatic modular design of robot swarms
Source: PeerJ Comput Sci. 2020 Dec 7;6:e322. doi: 10.7717/peerj-cs.322 (PMC7924708; doi:10.7717/peerj-cs.322)
Supplement: Supplemental Information 3 [file peerj-cs-06-322-s003.zip › argos3/doc/api/standalone/a00577_source.html]

ARGoS: plugins/simulator/entities/light\_entity.h Source File


- Main Page
- Related Pages
- Namespaces
- Classes
- Files

- File List
- File Members

# plugins/simulator/entities/light\_entity.h

Go to the documentation of this file.

```
00001 
00007 #ifndef LIGHT_ENTITY_H
00008 #define LIGHT_ENTITY_H
00009 
00010 namespace argos {
00011    class CLightEntity;
00012    class CLedEquippedEntity;
00013 }
00014 
00015 #include <argos3/core/simulator/entity/positional_entity.h>
00016 #include <argos3/plugins/simulator/entities/led_equipped_entity.h>
00017 
00018 namespace argos {
00019 
00020    class CLightEntity : public CLEDEntity {
00021 
00022    public:
00023 
00024       ENABLE_VTABLE();
00025 
00026    public:
00027 
00028       CLightEntity();
00029       
00030       CLightEntity(const std::string& str_id,
00031                    const CVector3& c_position,
00032                    const CColor& c_color,
00033                    Real f_intensity);
00034 
00035       virtual void Init(TConfigurationNode& t_tree);
00036 
00037       inline Real GetIntensity() const {
00038          return m_fIntensity;
00039       }
00040 
00041       inline void SetIntensity(Real f_intensity) {
00042          m_fIntensity = f_intensity;
00043       }
00044 
00045       virtual std::string GetTypeDescription() const {
00046          return "light";
00047       }
00048 
00049    protected:
00050 
00051       Real m_fIntensity;
00052    };
00053 
00054 }
00055 
00056 #endif
```

---

Generated on 10 Jul 2018 for ARGoS by 
 1.6.1
